# 3D Printing PLA/Gingival Stem Cells/ EVs Upregulate miR-2861 and -210 during Osteoangiogenesis Commitment

**DOI:** 10.3390/ijms20133256

**Published:** 2019-07-02

**Authors:** Jacopo Pizzicannella, Francesca Diomede, Agnese Gugliandolo, Luigi Chiricosta, Placido Bramanti, Ilaria Merciaro, Tiziana Orsini, Emanuela Mazzon, Oriana Trubiani

**Affiliations:** 1 Department of Medical, Oral and Biotechnological Sciences, University “G. d’Annunzio”, 66100 Chieti-Pescara, Italy; 2 IRCCS Centro Neurolesi “Bonino Pulejo”, 98124 Messina, Italy; 3 CNR-National Research Council, Institute of Cell Biology and Neurobiology (IBCN), Monterotondo, 00015 Roma, Italy

**Keywords:** microRNA, osteogenesis, angiogenesis, mesenchymal stem cells, extracellular vesicles, scaffold

## Abstract

Bone tissue regeneration strategies require approaches that provide an osteogenic and angiogenic microenvironment able to drive the bone growth. Recently, the development of 3D printing biomaterials, including poly(lactide) (3D-PLA), enriched with mesenchymal stem cells (MSCs) and/or their derivatives, such as extracellular vesicles (EVs) has been achieving promising results. In this study, in vitro results showed an increased expression of osteogenic and angiogenic markers, as RUNX2, VEGFA, OPN and COL1A1 in the living construct 3D-PLA/human Gingival MSCs (hGMSCs)/EVs. Considering that EVs carry and transfer proteins, mRNA and microRNA into target cells, we evaluated miR-2861 and miR-210 expression related to osteoangiogenesis commitment. Histological examination of rats implanted with 3D-PLA/hGMSCs/EVs evidenced the activation of bone regeneration and of the vascularization process, confirmed also by MicroCT. In synthesis, an upregulation of miR-2861 and -210 other than RUNX2, VEGFA, OPN and COL1A1 was evident in cells cultured in the presence of the biomaterial and EVs. Then, these results evidenced that EVs may enhance bone regeneration in calvaria defects, in association with an enhanced vascularization offering a novel regulatory system in the osteoangiogenesis evolution. The application of new strategies to improve biomaterial engraftment is of great interest in the regenerative medicine and can represent a way to promote bone regeneration.

## 1. Introduction

The body is unable to repair and regenerate large area bone defects afterwards trauma, infection, surgical resections, and other systemic problems exert negative effects on the bone healing process [1]. Starting from this consideration, the engineered tissue with different scaffolds for osteogenic repair has become one of the intriguing research fields over the past few years. Biomimetically designed materials matching the chemical and mechanical properties of tissue represent the best choice to favor mesenchymal stem cell (MSC) adhesion in the regenerative processes. In any case, cell-specific attachment and uniform cell distribution within the interior of 3D scaffold remain key challenges in healing critical sized defects. For the clinical use, these scaffolds must have some fundamental features including biocompatibility, biodegradability, mechanical strength, and matrix properties. Moreover, a key role is reserved to fiber and pore sizes that may influence some cellular responses, including migration, proliferation, and differentiation [2,3]. For these reasons, new biomaterials as bone substitutes able to induce minimal or no immune response and for encouraging implant/tissue interaction have been introduced. In particular, poly(ε-caprolactone), poly(glycolic acid) and poly(lactide) (PLA), including their copolymers, are among the most widespread synthetic biomaterials, characterized also by their biodegradability [4]. Among these, PLA is widely used in the field of regenerative medicine thanks to its good features, such as biodegradability, biocompatibility, thermal plasticity, and suitable mechanical effects [5].

Human dental MSCs derived from gingiva represent a new tool for bone regeneration. In fact, they can be easily expanded and are able to differentiate into osteogenic cells and to grow on biocompatible biomaterials [6,7]. They express MSC surface markers, such as Oct3/4, Sox-2, SSEA-4, CD29, CD44, CD73, CD90 and CD105, and lacking the expression of CD34, CD14 and CD45. 

Extensive research has been done on the therapeutic efficacy of MSCs [8], in particular for their protective functions, including immunomodulation process, in a paracrine manner by synthesis and secretion of a variety of cytokines, and growth factors [9,10]. Many studies have explored the potential beneficial applications of MSCs-conditioned media in different pathological models with the ability to regenerate neural, osteogenic, and myocardial cells [11,12].

Increasing evidence show that extracellular vesicles (EVs), which include a heterogeneous pool of membranous structures secreted by the majority of cells, can serve as powerful tools for cell-free therapy due to precise multifunctional molecular cargoes [13]. EVs contain functional proteins, lipids, and nucleic acids, such as mRNA and microRNA (miRNA) [14,15]. Qin et al. showed that EVs obtained from human MSCs are able to enter the osteoblasts and to deliver osteogenic miRNA by endocytosis, in this way regulating the osteogenic gene expression. Furthermore, the EVs were shown to promote bone regeneration in Sprague-Dawley rats subjected to calvarial defects [16] or to improve fracture healing in a mouse model [17], other than to show a strong proangiogenic induction [18].

In previous works, our group has already demonstrated the bone regenerative capacity of different scaffolds enriched with conditioned medium (CM) and the upregulation of vascular endothelial growth factor (VEGF) secretion and miR-210 expression in cells seeded on cancellous bovine bone [19,20]. The positive role of VEGF on osteogenesis has already been demonstrated [21]. In fact, vascularization is a fundamental process during osteogenesis and bone regeneration focusing the important role of VEGF during bone repair [22]. Moreover, during bone formation, miR-2861 present a positive regulatory role targeting Homeobox A2 (Hoxa2) and histone deacetylases (HDACs) respectively and indirectly favors the increase of Runt-related transcription factor-2 (RUNX2) [23]. Moreover, osteopontin (OPN) is a highly phosphorylated glycoprotein, which is a prominent component of the mineralized extracellular matrix of bone, and it showed an essential role for the secretion of type I collagen (COL1A1).

In the present study, we evaluated the regeneration of calvaria in rats transplanted with 3D printing PLA scaffold enriched with hGMSCs and/or EVs. In particular, the expression of molecules associated with osteoangiogenesis processes as miR-2861 and miR-210 together to RUNX2, VEGF, OPN and COL1A1 protein levels have been investigated. 

## 2. Results

### 2.1. 3D-PLA Evaluation

**Figure 1:**
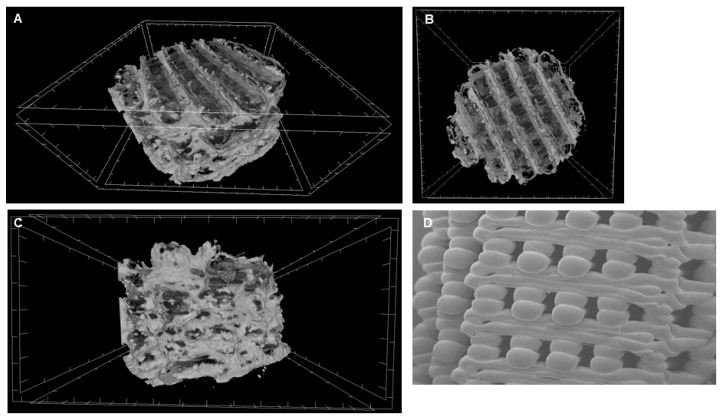
Biomaterial structure. Representative MicroCT pictures. (**A**) 3D viewing. (**B**) Transversal viewing. (**C**) Coronal viewing. (**D**) SEM acquisition of 3D-PLA (50× magnification).

3D-PLA has been analysed at MicroCT to define dimension features (Table 1) and observed via scanning electron microscopy (SEM) to define the surface morphology (Figure 1). At 50× magnification modular morphology structure is clearly visible (Figure 1).

### 2.2. Cell Characterization

**Figure 2:**
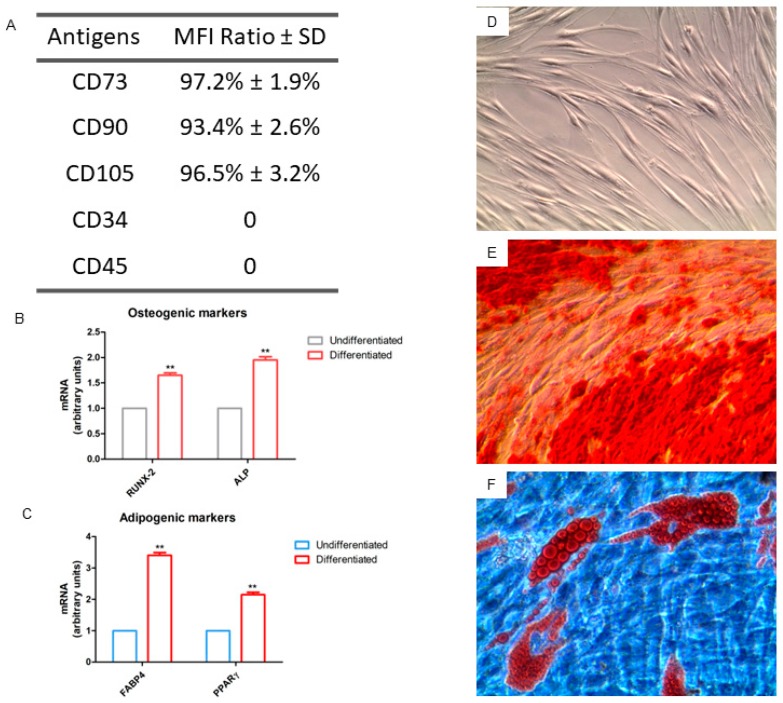
Cell characterization. (**A**) Flow cytometry detection of hGMSCs. (**B**) RT-PCR of osteogenic related markers: RUNX2 and ALP. (**C**) RT-PCR of adipogenic related markers: FABP4 and PPARγ. (**D**) Plastic adherent hGMSCs observed at light microscopy. (**E**) Osteogenic differentiation stained with Alizarin Red S solution showing calcium deposits. (**F**) Adipogenic differentiation stained with Oil Red O solution showing lipid droplets at cytoplasmic level. Mag: 10×. **p < 0.01.

The phenotypic profile of the hGMSCs, revealed through flow cytometry, showed that cells were positive for CD73, CD90 and CD105, while they were negative for CD34 and CD45, excluding the possibility that these are hematopoietic cells (Figure 2A). Cells were observed at inverted light microscopy, and they adhered to plastic substrate and possessed a fibroblastoid shape (Figure 2D). The hGMSCs demonstrated the potential of multi-lineage differentiation after being induced in a specific medium in vitro. Figure 2E and F showed the osteogenic and adipogenic differentiation. RT-PCR confirmed the light microscopy data showing an over expression in genes related to the ostegenesis and adipogenesis when compared to hGMSCs maintained under standard culture conditions (Figure 2B and C).

### 2.3. EVs Characterization

**Figure 3:**
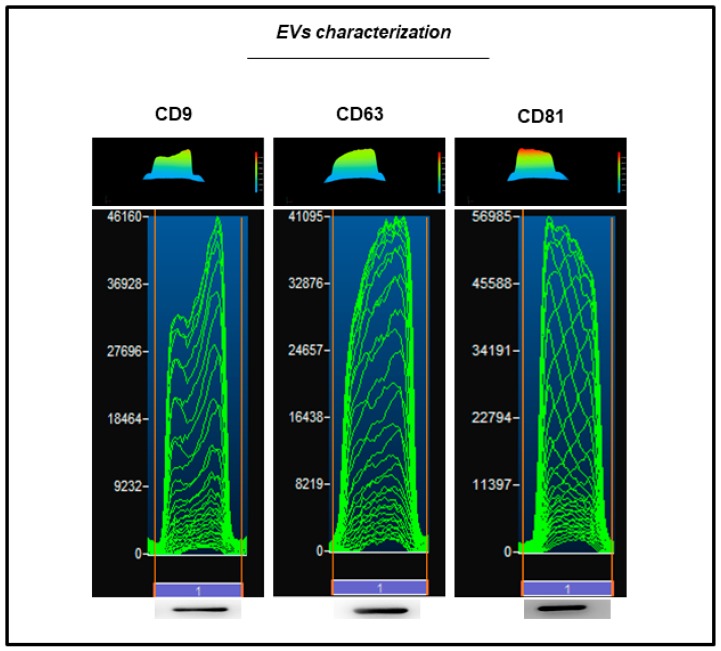
EVs characterization. Western blot showed the positivity for CD9, CD63 and CD81.

EVs collected from hGMSCs showed a positivity for CD9, CD63 and CD81 through the detection of protein levels at western blot analysis (Figure 3).

### 2.4. In Vitro Osteogenic Characterization

**Figure 4:**
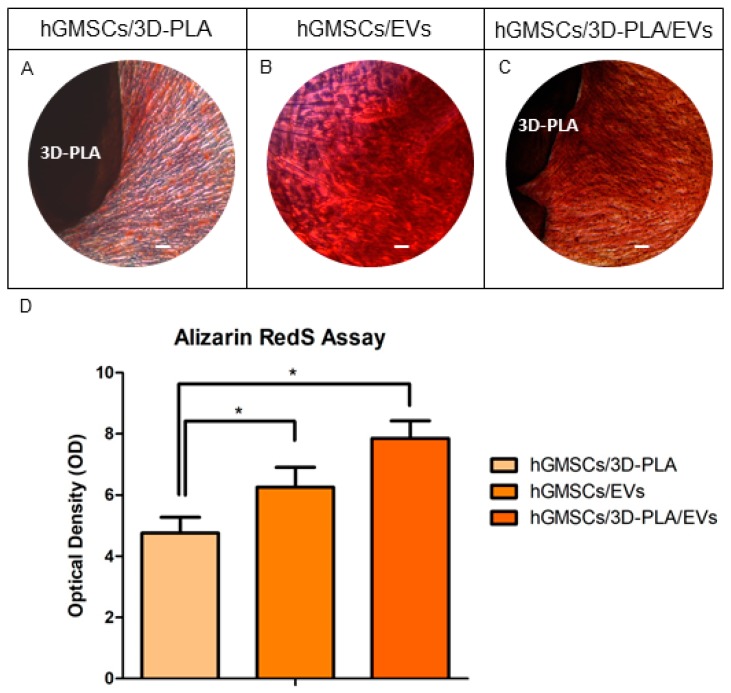
In vitro osteogenic experiments. Alizarin red S staining of hGMSCs cultured with 3D-PLA (**A**), with EVs (**B**) and with 3D-PLA/EVs (**C**) Mag: 10×; scale bar: 10 µm. Bar graph (**D**) showed the densitometric analysis of alizarin staining to quantify the different performances on osteogenic induction. *p < 0.05.

Light microscopy pictures were used to identify the calcium deposits and the extracellular matrix (ECM) mineralization by means Alizarin Red S staining in all samples (Figure 4A–C); the best results were obtained in hGMSCs cultured with 3D-PLA/EVs (Figure 4C). However, hGMSCs/EVs showed a better performance compared to hGMSCs/3D-PLA. Data were quantified using spectrometric analysis after 28 days of culture in basal medium (Figure 4D).

### 2.5. Gene Expression of Osteogenic Markers In Vitro

**Figure 5:**
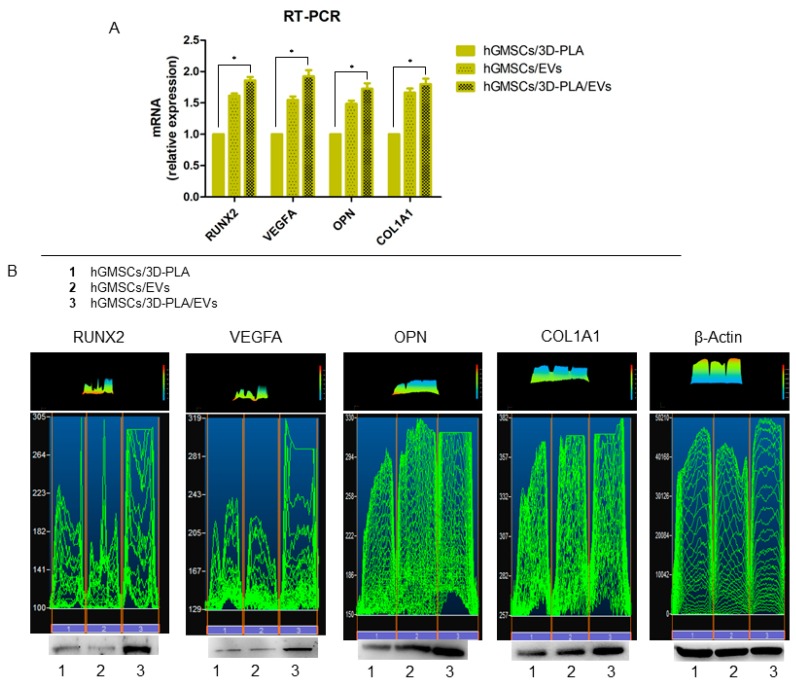
RUNX2 and VEGFA expression. (**A**) RT-PCR showed the different mRNA expression in hGMSCs/3D-PLA, hGMSCs/EVs and hGMSCs/3D-PLA/EVs. (**B**) Western blot analysis of protein expression: RUNX2, VEGFA, OPN and COL1A1. *p < 0.05.

RNA was extracted from cells cultured for 28 days in different conditions (hGMSCs/3D-PLA, hGMSCs/EVs and hGMSCs/3D-PLA/EVs) and subjected to real time RT-PCR to assess for changes in the expression of specific genes: RUNX2, VEGFA, OPN and COL1A1 are well known as markers involved in osteogenic process. As shown in Figure 5, RUNX2, VEGFA OPN and COL1A1 were increased in hGMSCs/3D-PLA/EVs compared with the hGMSCs/3D-PLA group. 

### 2.6. Western Blot Analysis of RUNX2 and VEGFA 

Given the important role played by RUNX2, VEGFA OPN and COL1A1 in bone regeneration, we evaluated the levels of these proteins in hGMSCs cultured with 3D-PLA and/or EVs in vitro. Western blot results showed a significant up regulation of RUNX2, VEGFA OPN and COL1A1 in hGMSCs/3D-PLA/EVs when compared to hGMSCs/3D-PLA and hGMSCs/EVs (Figure 5B). Beta actin has been used as internal control.

### 2.7. Micro-RNAs Expression

**Figure 6:**
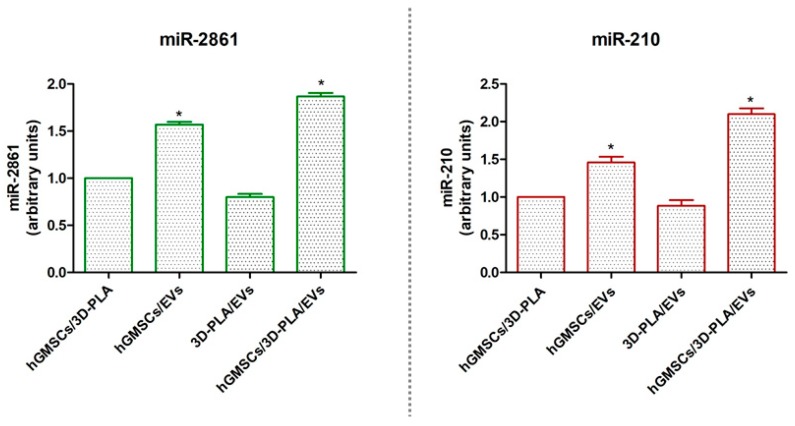
MiRNAs expression. Graphs showed the expression of miR-2861 and miR-210 after 28 days of culture in standard conditions. *p < 0.05.

RT-PCR showed an upregulation of miR-2861 and miR-210 in hGMSCs/3D-PLA/EVs samples when compared to the control (hGMSCs/3D-PLA). The same trend has been shown in hGMSCs/EVs although with low difference (Figure 6).

### 2.8. Histological Evaluation

**Figure 7:**
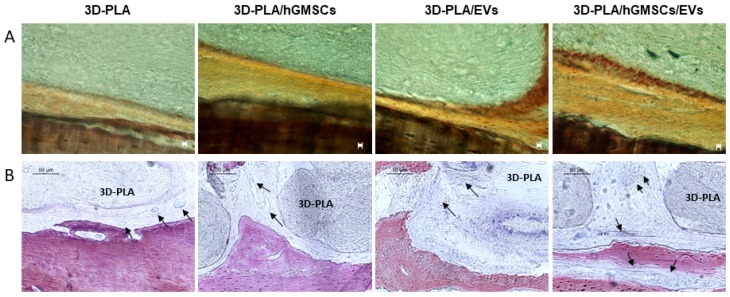
Histological evaluation in vivo. After six weeks of grafting, samples were stained with von Kossa silver staining (**A**) or Methylene blue and acid fuchsin images (**B**). (**A**) Images showed von Kossa positive staining in 3D-PLA/hGMSCs/EVs. Mag: 10×; scale bar: 10 µm. (**B**) The images indicated a higher vascularization in 3D-PLA/hGMSCs/EVs. Black arrows indicated blood vessels. Scale bar: 50 µm.

Histological assessment on the undecalcified calvaria were carried out after six weeks of grafting. 3D-PLA/hGMSCs/EVs showed a higher positive staining for calcium compared to 3D-PLA/hGMSCs and 3D-PLA/EVs. Section of 3D-PLA was negative (Figure 7A).

Staining with methylene blue and fuchsine acid solutions evidenced the increased vascularization in 3D-PLA/hGMSCs/EVs (Figure 7B).

### 2.9. MicroCT

**Figure 8:**
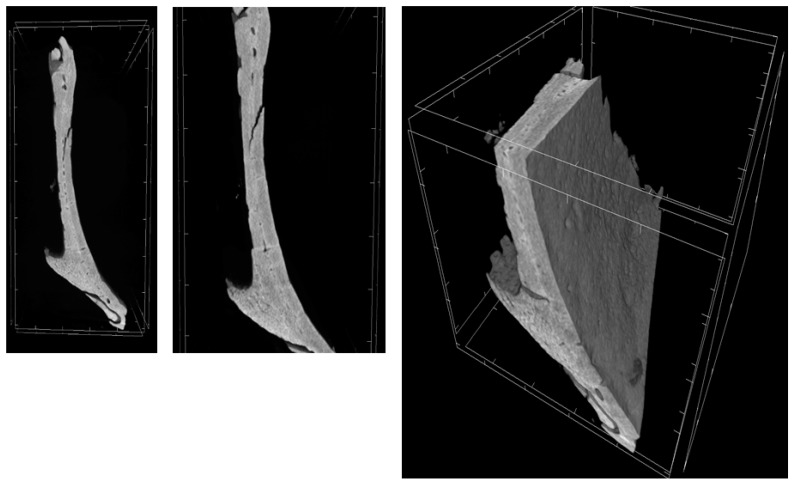
3D-MicroCT analysis. Three-dimensional volume rendering and virtual transverse sectioning of 3D-PLA.

**Figure 9:**
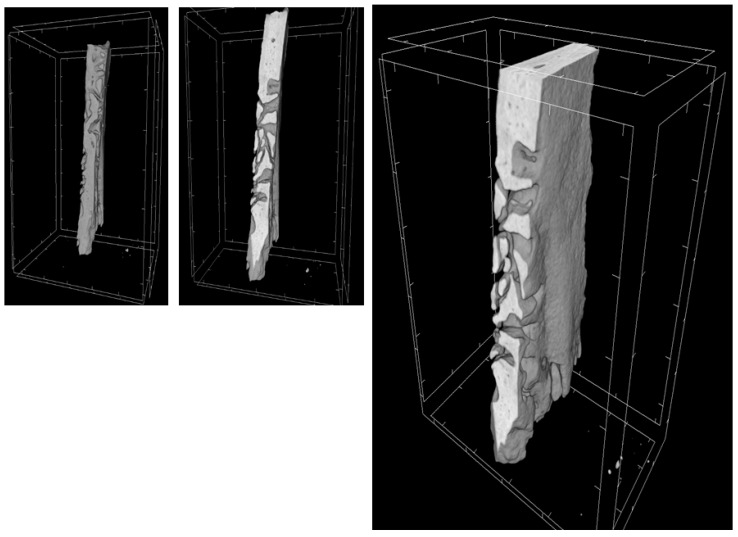
3D-MicroCT analysis. Three-dimensional volume rendering and virtual transverse sectioning of 3D-PLA/hGMSCs.

**Figure 10:**
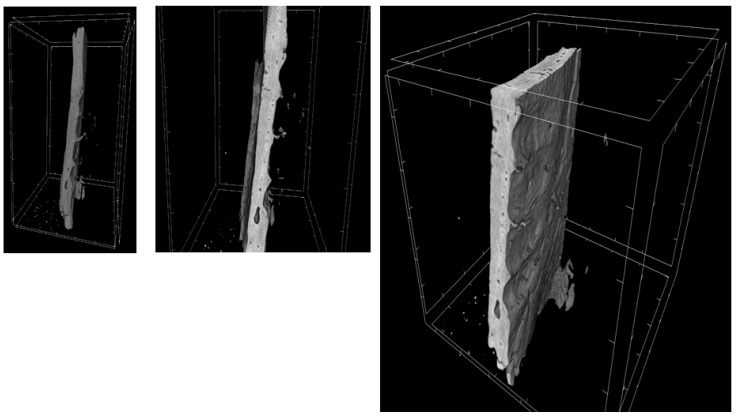
3D-MicroCT analysis. Three-dimensional volume rendering and virtual transverse sectioning of 3D-PLA/EVs.

**Figure 11:**
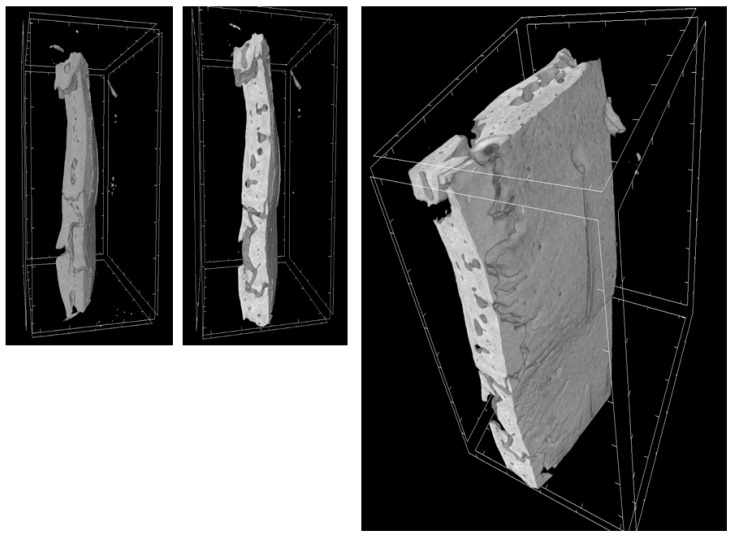
3D-MicroCT analysis. Three-dimensional volume rendering and virtual transverse sectioning of 3D-PLA/hGMSCs/EVs.

Through a three-dimensional virtual analysis performed with X-ray Micro-tomography (Figure 8, Figure 9, Figure 10 and Figure 11), a high rate of regeneration and integration level was observed in 3D-PLA/hGMSCs/EVs (Figure 11), with a strong resemblance to naive bone, when compared with 3D-PLA (Figure 8), 3D-PLA/hGMSCs (Figure 9) and 3D-PLA/EVs (Figure 10). 

The quantification of bone parameters supported the results of the MicroCT images, as shown in Table 2.

## 3. Discussion

3D printing is an attractive technique to fabricate customized, feasibly and economically advantageous scaffolds and devices for tissue engineering applications [24]. Scaffold’s performance is related to chemistry, pore size, pore volume and mechanical strength. For bone tissue regeneration, interconnected porosity is essential to facilitate nutrients and molecule transport into the scaffold for cell proliferation and subsequent vascularization. PLA is an absorbable polymer that has often been used in skeletal tissue engineering, which is able to offer mechanical stability other than adequate cellular migration and provide nutrients after in vivo implantation [20]. In a previous study, our group tested different scaffold designs with different porosity and filament dimension evaluating in vitro degradation and the cytotoxicity of degradation byproducts [25]. In this work, the SEM image showed the modular morphology of the 3D-PLA.

Most of bone tissue engineering strategies are based on biocompatible scaffolds seeded with tissue-specific cells in particular MSCs. The therapeutic ability of MSCs has been attributed at their key mechanisms as homing process, differentiation process, and secretion of bioactive molecules [18]. Even if many studies are focused on the regenerative ability of MSCs, a particular attention is directed to the secretion cell products, in particular on the secretome and EVs [26]. Released membrane vesicles from eukaryotic cells, as exosomes, microparticles, microvesicles, and apoptotic bodies, can be retained as a dynamic extracellular vesicular compartment, strategic for their paracrine or autocrine biological effects on tissue metabolism [27], since their cargo is composed of different proteins, mRNA and microRNA that act on different target cells. For this reason, due to this shipping of information, EVs are retained to be the most promising therapeutic tool for multiple diseases. EVs obtained from hGMSCs are positive for CD9, CD63, CD81 and tumor suppressor gene (TSG101), and the dynamic light scattering (DLS) analysis showed the presence of a heterogeneous population of EVs, with sizes from 100 to 1200 nm [25]. In this work, the analysis of calcium deposition in vitro and ECM mineralization using Alizarin Red S evidenced a better osteogenic performance of hGMSCs cultured in the presence of EVs compared to 3D-PLA. This result may indicate that most of the achievements are due to EVs stimulus. However, the combination of both EVs and 3D-PLA showed the best osteogenic performance. This result is also confirmed by the increased gene expression and protein levels of the osteogenic marker RUNX2, OPN and COL1A1 in 3D-PLA/hGMSCs/EVs.

The development of a vascular system for the delivery of oxygen and nutrients represents a key event in tissue repair. In particular, bones are highly vascularized tissues and it is clear that osteogenesis and angiogenesis are two processes intensely linked. Vascularization is a fundamental process during osteogenesis, blood vessels play a role as transporters of growth factors, minerals and others into the osteogenic microenvironment [28]. Osteoblasts are able to produce pro-angiogenic factors, including VEGF [29]. The VEGF family is composed of different members, but VEGFA, commonly named VEGF, was the first member to be detected and have a special position in angiogenesis. It has been described that neural crest cells produce high quantity of VEGF, and moreover, the VEGF deletion causes calvarial and mandibular malformations [30]. In this work, we evidenced that both gene expression and protein level of VEGF were increased in 3D-PLA/hGMSCs/EVs in vitro. This result can suggest that the enrichment of 3D-PLA with EVs may increase angiogenesis other than osteogenesis.

EV-transported miRNA transferring between cells has been proposed to be a mechanism for intercellular signaling [31]. MicroRNAs have been extensively studied in the regulation of many cellular processes, including proliferation, apoptosis, metabolism, neuronal patterning and tumorigenesis [32]. MiRNAs are also involved in stem-cell functions, such as differentiation, by controlling the post-transcriptional process and additionally play a key role in transduction angiogenic signals [33]. Our in vitro results evidenced the upregulation of miR-2861 and miR-210 in hGMSCs/EVs and a further increase of both miRNA was found in 3D-PLA/hGMSCs/EVs. It is already known that EVs derived from MSCs contained miR-210 and that it exerts a pro-angiogenic effect [34].

In particular, miR-210 is involved in the inhibition of the expression of tumor suppressive genes and in the induction of cell proliferation [19]. Recent evidence indicates that miR-210 plays a critical role in cell survival and angiogenesis [35]. 

Upregulated expression of miR-210 was detected in bone morphogenetic protein 4 (BMP-4)-induced osteoblastic differentiation and miR-210 inactivation decreases the ability of HUVEC cells to form capillary-like structures and migrate in response to VEGF [36]. miR-210 is implicated in promoting osteoblast differentiation by increasing VEGF, alkaline phosphatase (ALP) and osterix (OSX) expression in rat MSCs and suppressed adipocyte differentiation, due to a decrease of Peroxisome Proliferator-Activated Receptor γ (PPARɣ) in vitro [37]. Moreover, oral stem cells seeded in the presence of biomaterial showed that the miR-210 up regulation was associated to the release VEGFA in the culture medium in an exponential manner [19]. In a previous study, our group found that the osteogenic differentiation was greater in dental MSCs grown onto 3D scaffold in osteoinductive conditions associated to the overexpression of miR-2861 and RUNX2 [38]. RUNX2/miR-3960/miR-2861 positive feedback loop is responsible for osteoblast differentiation [39], in addition to the induction of genes essential for osteoblast differentiation, RUNX2 transactivates miR-3960/miR-2861. In succession, miR-3960 and miR-2861 preserve the levels of RUNX2 mRNA and protein via repressing Hoxa2 and HDAC5, and in this way they stabilize the osteoblast differentiation [39]. The results of this study are in line with the previous ones. Indeed, the upregulation of miR-2861 and miR-210 was associated with increased VEGF and RUNX2 expression and osteogenic differentiation. The upregulation of both miRNA may be responsible of the increased osteogenesis and angiogenesis. These data indicated a fundamental role of EVs that, thanks to their miRNA content, play a main role in the osteoangiogenic process.

We have analyzed the behavior of the different living constructs in vivo considering the bone angiogenesis induction. The better osteoangiogenic performance of 3D-PLA/hGMSCs/EVs was also confirmed in vivo in rats subjected to a calvaria damage. Indeed, the histological analysis evidenced the presence of osteoangiogenesis processes in the group 3D-PLA/hGMSCs/EVs. This group showed the best performance in terms of bone regeneration confirmed also by MicroCT analysis. 

In order to maintain cell viability and differentiation in in vivo experiments after bone grafting, it is required the development of blood vessel network to perfuse oxygen and nutrient to avoid cell death [40].

## 4. Materials and Methods

### 4.1. Scaffold Material

PLA material samples were developed as previously reported by Diomede et al [25]. A commercial CAD software was used (Rhinoceros 5, McNeel Europe, Barcelona, Spain). Briefly, the projects were applied to a printing slicing software (Cura 15.04, Ultimaker B.V., Geldermalsen, The Netherlands) and then the sliced project was transferred to a commercial fuse filament fabrication 3D printer (DeltaWASP 2040; CSP srl, Massa Lombarda, Italy). The printed constructs obtained were ready for the following experiments. 

### 4.2. In Vitro Study

#### 4.2.1. Ethics Statement for In Vitro Experiments

The study was performed after the collection of the written approval from the Medical Ethics Committee at the Medical School, “G. d’Annunzio” University, Chieti, Italy (n°266 17 April 2014, Principal Investigator: Trubiani Oriana). Before sample collection, the written informed consent was obtained from all enrolled subjects. Both the Department of Medical, Oral and Biotechnological Sciences as well as the Laboratory of Stem Cells and Regenerative Medicine are certified in accordance with the quality standard ISO 9001:2008 RINA (certificate no. 32031/15/S). The study was also conducted under the Helsinki Declaration guidelines (2013). 

#### 4.2.2. Cell Culture Establishment and Characterization

Human gingival tissue biopsies were performed in patients scheduled to remove gingival tissues during surgical procedure in teeth scheduled to remove for orthodontic purpose. After, cells were cultured using the chemically defined MSCGM-CD ™ BulletKit media (MSCGM-CD) (Lonza, Basel, Switzerland), that was changed twice a week. For the following experiments, cells at passage 2 were used. Cell characterization and multi-lineage differentiation was performed as previously described [26].

#### 4.2.3. Scanning Electron Microscopy Analysis

Zeiss Evo50 SEM (Zeiss, Jena, Germany) was used to acquire SEM images. 3D-PLA samples were covered by a layer of sputtered gold by means the Emitech K550 (Emitech Ltd., Ashford, UK) sputtering apparatus [41].

#### 4.2.4. Extracellular Vesicles (EVs) Isolation

hGMSCs at second passage were cultured at a density of 15 × 10^3^/cm^2^. After 48 h of incubation, the CM was harvested and centrifuged at 3000× *g* for 15 min in order to remove cells in suspension and cell debris [42]. The Exoquick TC commercial kit (System Biosciences, Palo Alto, CA, USA) was used for the EVs extraction. Briefly, 2 mL of ExoQuick TC were mixed with 10 mL of CM obtained from hGMSCs. The mix obtained was then incubated overnight at 4  °C without rotation. After, the mix was centrifugated at 1500× g for 30 min in order to sediment the EVs. The pellets were resuspended in 200 μL of phosphate-buffered saline (PBS). The EVs, split in two aliquots, were precipitated, and the quantification of whole homogenate proteins was carried out to confirm the presence of the release of EVs by hGMSCs. To characterize the EVs, western blotting analysis has been performed as following described.

#### 4.2.5. Scaffold Preparation

3D-PLA was cut into small pieces of about 4 × 7 mm^2^, cell culture medium was added and it was maintained for 24 h at 37 °C in order to verify the sterility. After 24 h, 2000 cells/scaffold were seeded onto the scaffold. In order to make the scaffolds enriched with EVs, EVs were added after 24 h at the concentration 0.5 µg/µL into 3D-PLA or 3D-PLA/hGMSCs. 

#### 4.2.6. In Vitro Osteogenesis Performance

hGMSCs were seeded in the presence of 3D-PLA, EVs and 3D-PLA/EVs as reported in the previous paragraph. The evaluation of calcium deposition and ECM mineralization was performed after 28 days of culture using Alizarin Red S staining assay. Cells were fixed in 10% (*v/v*) formaldehyde (Sigma-Aldrich, Milan, Italy) for 30 min. Afterwards, cells were treated with 0.5% Alizarin Red S in H_2_O, pH 4.0, for 1 h at room temperature. In order to perform staining quantification, 800 μL 10% (*v/v*) acetic acid was added to each well. After an incubation of 30 min, cells were scraped from the plate and were then moved into a 1.5-mL vial. The obtained suspension, overlaid with 500 μL mineral oil (Sigma-Aldrich), was heated to 85 °C for 10 min. After, the suspension was transferred to ice for 5 min and centrifuged at 20,000× *g* for 15 min. 500 μL of the supernatant were transferred into a new 1.5-mL vial and 200 μL of 10% (*v/v*) ammonium hydroxide were added (pH 4.1–4.5); 150 μL of the supernatant obtained from cultures were read in triplicate at 405 nm by a spectrophotometer (Synergy HT, BioTek, Bad Friedrichshall, Germany).

#### 4.2.7. RNA Isolation and Real Time-PCR Analysis

Total RNA was isolated from cells seeded with three different samples: hGMSCs/3D-PLA, hGMSCs/EVs and hGMSCs/3D-PLA/EVs after 28 days of culture. RNA isolation was performed using Total RNA Purification Kit (NorgenBiotek Corp., Ontario, CA, USA) following the manufacturer’s instructions. cDNA was obtained using the M-MLV Reverse Transcriptase reagents (Applied Biosystems, Foster City, CA, USA). Real-time PCR was performed with the Mastercycler ep realplex real-time PCR system (Eppendorf, Hamburg, Germany). hGMSCs expression of RUNX2 and VEGFA was evaluated after 28 days of culture. Gene expression assays was performed as previously described [43]. Commercially available TaqMan Gene-Expression Assays (RUNX2: Hs00231692_m1; VEGFA: Hs00900055_m1; OPN: Hs00959010_m1; COL1A1: Hs00164004_m1) and the TaqMan Universal PCR Master Mix (Applied Biosystems) were used according to standard protocols. Beta-2 microglobulin (B2M Hs99999907_m1) (Applied Biosystems) was used for template normalization. RT-PCR was performed in three independent experiments, duplicate determinations were carried out for each sample.

#### 4.2.8. Western Blot Analysis 

Proteins were collected from hGMSCs/3D-PLA, hGMSCs/EVs and hGMSCs/3D-PLA/EVs samples (40 µg/sample) after 28 days of culture. The western blot procedure was performed as previously reported. RUNX2 (Santa Cruz Biotechnology, Santa Cruz, CA, USA; 1:1000) and VEGFA (Santa Cruz Biotechnology; 1:1000) were used as primary antibody. β-Actin (Santa Cruz Biotechnology; 1:750) was used to assess the uniform protein loading. To characterize the EVs, CD9 (SantaCruz Biotechnology; 1:500), CD63 (Abcam, Cambridge, UK; 1:500) and CD81 (Santa Cruz Biotechnology; 1:500) were used as the primary antibody. Bands were analyzed by the ECL method using Alliance 2.7 (UVItec Limited, Cambridge, UK). 

#### 4.2.9. MicroRNAs Quantization

miRNA were extracted after 28 days of culture using the PureLink RNA mini kit (Life Technologies, Milan, Italy), treated with the RNase-Free DNase Set (Qiagen, Venlo, The Netherland) according to the instructions of the manufacturer and quantified with Nanodrop2000 (Thermo-Scientific, Waltham, MA, USA). Gene sequences were from NCBI (http://www.ncbi.nlm.nih.gov), and RNA sequences for miR-286 and miR-210 were used into the Universal ProbeLibrary (UPL) Assay Design Center software (https://www.rocheappliedscience.com) to identify primers and UPL probe. Total RNA (50–200 ng) was retrotranscribed with High-Capacity cDNA Reverse-Transcription Kit (Life Technologies). MicroRNA quantization was performed using stem-loop RT primers designed with a modification to include the UPL #21 sequence-binding site [38]. UPL probe #21 was from the UPL database (Roche Diagnostics, Basel, Switzerland). Total RNA (50 ng) was retrotranscribed with a TaqMan MicroRNA Reverse-Transcription Kit (Life Technologies). Reactions were incubated for 30 min at 16 1 _C, followed by pulsed RT of 60 cycles at 30 1 °C for 30 s, 42 1 °C for 30 s, and 50 1 °C for 1 s. Real-time PCRs were performed in an Applied Biosystems 7900 instrument. miRNA and mRNA levels were measured using Ct (threshold cycle). The target amount, normalized to endogenous reference 18S/RNU44 and relative to a calibrator, was given by 2 DDCt and/or 2 DCt methods (Life Technologies).

### 4.3. In Vivo Study

#### 4.3.1. Animals

Male Wistar rats, acquired from Harlan Milan, Italy, were used in this experiment (weight 300–350 g). Animals were housed in individually ventilated cages and maintained under 12 h light/dark cycles, at 21 ± 1 °C and 50–55% humidity with food and water ad libitum.

#### 4.3.2. Ethics Statement for Animal Use 

All animal care and use was performed in accordance to the European Organization Guidelines for Animal Welfare. The study was authorized by the Ministry of Health “General Direction of animal health and veterinary drug” (Authorization 768/2016-PR 28/07/2016- D.lgs 26/2014). The experiments were designed in order to minimize the total number of animals needed.

#### 4.3.3. Scaffold Grafting

Rats were anesthetized with a mixture of tiletamine and xylazine (10 mL/kg, intraperitoneal; i.p.) and the implant site was disinfected using iodopovinone (Betadine). After trichotomy, a median sagittal incision of about 1.0 cm in the frontoparietal region, a total thickness cut was applied. The calvaria was exposed and a circular section of the bone receiving site, with a diameter of 5 mm and a height of 0.25 mm, was damaged using a rotary instrument at a controlled speed (trephine milling machine, Alpha Bio-Tec, Siena, Italy) under constant irrigation of physiological solution.

Given their texture and flexibility 3D-PLA, 3D-PLA/hGMSCs, 3D-PLA/EVs and 3D-PLA/hGMSCs/EVs were put into contact with the bone in such a way to cover the damaged area. The skin flap was sutured using small absorbable sutures of reduced diameter (Caprosyn 6-0), with interrupted points. In the post-operative period, standard feeding and hydration were maintained constant. 

#### 4.3.4. Experimental Design

Rats were randomly divided into the following groups:

1. 3D-PLA (*N* = 4): rats subjected to calvaria bone damage and grafted with 3D-PLA;
2. 3D-PLA/hGMSCs (*N* =4): rats subjected to calvaria bone damage and grafted with 3D-COL enriched with hGMSCs;
3. 3D-PLA/EVs (*N* = 4): rats subjected to calvaria bone damage and grafted with 3D-PLA enriched with EVs;
4. 3D-PLA/hGMSCs/EVs (*N* = 4): rats subjected to calvaria bone damage and grafted with 3D-PLA enriched with hGMSCs and EVs;

After six weeks, the animals were euthanized by intravenous administration of Tanax (5 mL/kg body weight) and their calvariae were processed for morphological analysis. 

#### 4.3.5. Histological Evaluation

In order to perform histological analysis, samples were fixed for 72 h in 10 % formalin solution, dehydrated in ascending graded alcohols and embedded in LR White resin (Sigma-Aldrich) [44]. After polymerization, undecalcified oriented cut sections of 50 μm were obtained and after ground down to about 30 μm using the TT System (TMA2, Grottammare, Italy). Sections were washed three times with distilled water and placed in silver nitrate solution (1%) under intense light for 3 h. Then, silver nitrate solution was removed and the scaffolds were washed again three times with distilled water. By adding sodium thiosulfate solution (5%) for 5 min, unreacted silver was removed from the scaffolds. Finally, the samples were washed with distilled water and observed by invert microscopy. The investigation was carried out by means of a bright-field light microscope (Leica Microsystem, Milan, Italy) connected to a high-resolution digital camera DFC425B Leica (Leica Microsystem). 

In order to evaluate vascularization, the sections were observed under a light microscope after a double-staining procedure with methylene blue and fuchsin acid solutions. 

#### 4.3.6. MicroCT Evaluation

Virtual 3D analysis was performed through high-resolution X-ray Micro-Computed-Tomography (Micro-CT Skyscan 1172G Bruker, Kontich, Belgium). The acquisition of tomographic image datasets was obtained using 0.5 mm Al filter, image pixel/size of 7.4 um, camera binning 2 × 2, tube voltage peak of 49 kV, tube current of 200 uA, exposure time of 820 ms. The reconstructions of the acquired 2D images (about 1300 slices per sample) in volume images were performed using built-in NRecon Skyscan reconstruction software (Bruker software package, Version: 1.6.6.0). The volume rendering and the virtual sectioning views were generated using 3D Visualization Softwares CTvox v. 2.5 and DataViewer v. 1.4.4 (Skyscan Bruker software package). Data were analyzed using Bruker CT-Analyser software Version 1.13 (CTAn). A volume of interest (VOI) of 300 slides was extrapolated from each dataset, corresponding to the central zone and identical for each sample, starting from a ROI (region of interest) of 6 × 4 mm^2^, which included the damage for automated 3D measurements of bone parameters. The bone volume (BV), percent BV (BV/tissue volume, TV), bone surface (BS), bone specific surface (BS/BV) and bone surface density (BS/TV) were evaluated. Data were expressed as the mean ± SD values.

### 4.4. Data and Statistical Analysis

Data were expressed as means and standard deviation of the recorded values. Statistical analysis was performed using Kruskal-Wallis test followed by Dunn’s multiple comparison test. Differences were considered significant when *p* < 0.05.

## 5. Conclusions

Most of the improvements observed seem to depend on the EVs stimulus. Then, this work highlights the important role played by EVs during osteoangiogenesis commitment, explaining that one of the mechanisms associated with the regulation of osteogenic differentiation process is related to miRNAs expression. Based on this scenario, we believe that the combination of 3D printed PLA porous scaffolds enriched with hGMSCs and EVs is an efficient tool to promote the osteoangiogenesis, a pronounced complexity process, that has as immense potential as an adequate novel therapeutic strategy for bone tissue lesions in areas undergoing a severe injury, necrosis, infection, degeneration, and resection with an elevated profile of safety and effectiveness.

## Figures and Tables

**Table 1:** Characterization of 3D-PLA.

Fiber diameter	2.245 × 10^2^ µm
**Pore size**	5.042 × 10^5^ µm^2^
**Interconnectivity**	1877
**Surface area**	6 × 10^3^ µm^3^

**Table 2:** Morphometric analysis of MicroCT images.

MicroCT Parameter	3D-PLA	3D-PLA/hGMSCs	3D-PLA/EVs	3D-PLA/hGMSCs/EVs
**BV (µm^3^)**	3.2 × 10^9^ ± 1.6 × 10^8^	4.3 × 10^9^ ± 1.7 × 10^8^	3.4 × 10^9^ ± 2.9 × 10^8^	6.8 × 10^9^ ± 2.6 × 10^8^
**BV/TV (%)**	3.9 ± 0.1	5.2 ± 0.2	4.1 ± 0.3	8.2 ± 0.3
**BS (µm^2^)**	3.1 × 10^7^ ± 2.2 × 10^6^	3.2 × 10^7^ ± 2.4 × 10^6^	3.2 × 10^7^ ± 2.3 × 10^6^	6.6 × 10^7^ ± 1.7 × 10^6^
**BS/BV (1/µm)**	9.8 × 10^-3^ ± 3.8 × 10^-4^	7.4 × 10^-3^ ± 3.8 × 10^-4^	9.5 × 10^-3^ ± 3.9 × 10^-4^	9.8 × 10^-3^ ± 1.6 × 10^-4^
**BS/TV (1/µm)**	3.8 × 10^-4^ ± 1.3 × 10^-5^	3.9 × 10^-4^ ± 2.9 × 10^-5^	3.9 × 10^-4^ ± 2.6 × 10^-5^	8.1 × 10^-4^ ± 1.3 × 10^-5^

BV: bone volume, TV: trabecular volume; BS: bone surface. **BV**: 3D-PLA vs. 3D-PLA/hGMSCs/EVs **p < 0.01. **BV/TV**: 3D-PLA vs. 3D-PLA/hGMSCs/EVs **p < 0.01. **BS**: 3D-PLA vs. 3D-PLA/hGMSCs/EVs *p < 0.05. **BS/BV**: 3D-PLA vs. 3D-PLA/hGMSCs *p < 0.05. **BS/TV**: 3D-PLA vs. 3D-PLA/hGMSCs/EVs *p < 0.05.

## Abbreviations

MSCs	Mesenchymal stem cells
PLA	poly(lactide)
EVs	Extracellular vesicles
miRNA	microRNA
CM	conditioned medium
VEGF	Vascular endothelial growth factor
Hoxa2	Homeobox A2
HDACs	Histone deacetylases
RUNX2	Runt-related transcription factor-2
SEM	Scanning electron microscopy
ECM	Extracellular matrix
DLS	Dynamic light scattering
ALP	Alkaline phosphatase
OSX	Osterix
PPARγ	Peroxisome Proliferator-Activated Receptor γ
BMP-4	Bone morphogenetic protein 4
PBS	Phosphate-buffered saline
UPL	Universal ProbeLibrary
